# Multilocus genotyping of *Enterocytozoon bieneusi* derived from nonhuman primates in southwest China

**DOI:** 10.1371/journal.pone.0176926

**Published:** 2017-05-12

**Authors:** Zhijun Zhong, Wei Li, Lei Deng, Yuan Song, Kongju Wu, Yinan Tian, Xiangming Huang, Yanchun Hu, Hualin Fu, Yi Geng, Zhihua Ren, Guangneng Peng

**Affiliations:** 1 The Key Laboratory of Animal Disease and Human Health of Sichuan Province, College of Veterinary Medicine, Sichuan Agricultural University, Sichuan Province, China; 2 Chengdu Research Base of Giant Panda Breeding, Sichuan, China; Tulane University, UNITED STATES

## Abstract

*Enterocytozoon bieneusi* has been increasingly reported in non-human primates (NHPs) in recent years, and this has garnered attention. However, reports of *E*. *bieneusi* infections in NHPs are limited worldwide. To appreciate the genetic diversity and assess the zoonotic potential during the transmission of human microsporidiosis, we examined a total of 369 fecal samples from NHPs and performed PCR amplification of the ITS gene of *E*. *bieneusi*. An infection rate of 12.5% (46/369) was detected in NHPs, with three known genotypes (D, PigEBITS7, and SC02) and a novel genotype (SCM01) characterized. Phylogenetic analysis indicated that all four genotypes in our study were classified as zoonotic group 1. Multilocus genotyping of positive *E*. *bieneusi* strains revealed that 36, 37, 30, and 29 specimens were successfully amplified and sequenced to generate 16, six, four, and five types of MS1, MS3, MS4, and MS7 loci, respectively. Twenty-four specimens were successfully amplified and sequenced at all four loci, forming 13 multilocus genotypes (MLGs). The occurrence of zoonotic genotypes suggests that zoonotic transmission of *E*. *bieneusi* between humans and NHPs has probably occurred and NHPs could be a source of human microspordiosis.

## Introduction

Microsporidiosis, an emerging infectious disease, is caused by microspordia, leading to gastrointestinal disease in domestic animals, wildlife, and humans. *Enterocytozoon bieneusi* is one of the main human pathogenic microsporidian species [1]. Its predominant clinical manifestations include enteritis, cholangitis, cholecystitis, rhinitis, sinusitis, pneumonia, and enteritis, with symptoms including self-limiting diarrhea or life-threatening chronic diarrhea [2]. In 1985, *E*. *bieneusi* was identified in an AIDS patient, and this was the first report of a human infection caused by this species [3]. Subsequently, an increasing number of humans have been reported to be infected with *E*. *bieneusi* in developing and developed countries [4–6]. These humans include AIDS patients, HIV-uninfected people, organ transplant recipients, and immunocompetent individuals [7–11]. *E*. *bieneusi* can be transmitted to humans by anthroponotic or zoonotic transmission, after exposure to infected individuals or animals, respectively [12]. Because of its public significance and great potential threat to the public, microsporidia are on the NIAID Priority Pathogens List and are considered Category B pathogens [13].

A molecular method has been widely used to identify the occurrence and genotypes of *E*. *bieneusi*, and is generally based on the internal transcribed spacer (ITS) region of the rRNA, which has a high degree of diversity among strains [14]. To date, more than 240 genotypes have been characterized in various hosts. Phylogenetic analysis has classified these genotypes into nine groups enabling researchers to assess the zoonotic potential and host specificity. Specifically, *E*. *bieneusi* isolates that belong to group 1 have major zoonotic potential with broad host ranges, whereas *E*. *bieneusi* isolates in group 2 to 9 are host-adapted with little or no zoonotic potential [15]. The use of a single marker can somewhat explain the possible transmission routes in humans and animals and can possibly identify *E*. *bieneusi* genotypes, albeit relatively inaccurately [1, 16]. A multilocus sequence typing (MLST) tool employing three microsatellite loci (MS1, MS3, and MS7) and one minisatellite (MS4) locus was developed by Feng *et al* [17] and has a high-resolution for *E*. *bieneusi* genotyping. This tool can be used to better elucidate the possible transmission modes and determine the public health significance of *E*. *bieneusi* of animal origin, and identify the sources of human *E*. *bieneusi* infections.

Currently, *E*. *bieneusi* has been detected in both immunocompromised and immunocompetent NHPs and infections in these animals have been reported in several countries, including China, Central African Republic, Portugal, and Kenya [18–21]. In China, reports have shown *E*. *bieneusi* infections in some provinces, but very few studies have investigated the occurrence of *E*. *bieneusi* in Sichuan and Guiyang provinces, southwest of China. In our study, we examined the occurrence of *E*. *bieneusi* in NHPs and genotyped *E*. *bieneusi*-positive strains by ITS sequencing and MLST analysis. Furthermore, the potential role of NHPs in the zoonotic transmission of human microsporidiosis was evaluated.

## Methods

### Ethic statement

Before sample collection, all animal work was approved by the Institutional Animal Care and Use Committee of the Sichuan Agricultural University under permit number DYY-S20153801. Prior to the collection of fecal specimens from NHPs, permission was obtained from owners.

### Specimens

From January 2014 to November 2015, 369 fecal specimens from non-human primates were collected from Sichuan province and Guiyang province and separately stored in centrifuge tubes. Specifically, 303 samples were derived from rhesus macaques from Ya’an rhesus macaque base (102°51′E-103°12′E, 29°40′N-30°14′N), Guiyang zoo (106°21′E-106°53′E, 26°45′N-27°12′N), Mount Emei (103°10′E-103°37′E, 29°16′N-29°43′N) or natural environment in Baoxing (102°29′E-103°01′E, 30°09′N-30°56′N) (April 2014 to March 2015). Thirty samples were from short-tailed Tibetan macaques in Mount Emei (January 2014 to March 2014). Six specimens were from wild golden snub-nosed monkeys in Baoxing (April 2014). Thirty specimens were from northern white-cheeked gibbons from Guiyang zoo (July 2014 to January 2015) (Table 1). The samples were collected in sterile disposable plastic tubes. Samples were preserved in 2.5% potassium dichromate at 4°C in a refrigerator. All samples were processed within 24 hours of collection.

**Table 1:** Prevalence of *Enterocytozoon bieneusi* and genotypes in nonhuman primates based on PCR and sequence analysis of the ITS locus.

common name (scientific name)	Area	mode	No. tested	No. (%) of positive specimens	ITS genotypes
Rhesus macaque (*Macaca mulatta*)	Ya’an rhesus macaque base	captive	227	24 (10.6)	D (21), SC02 (3)
	Guiyang zoo	captive	16	1 (6.3)	D (1)
	Ya’an	wide	60	1 (1.7)	SCM01 (1)
Short-tailed tibetan macaque (*Macaca thibetana*)	Mout Emei	captive	30	0 (0)	
Golden snub-nosed mokey (*Rhinopithecus roxellanae*)	Ya’an	wide	6	0 (0)	
Northern white-cheeked gibbon (*Nomascus leucogenys*)	Guiyang zoo	captive	30	20 (66.7)	D (11), PigEBITS7 (9)
**Total**			369	46 (12.5)	D (33), PigEBITS7 (9), SC02 (3), SCM01 (1)

### DNA extraction and PCR amplification

Before extracting DNA, the fecal samples were washed with distilled water until the potassium dichromate was removed. Subsequently, genomic DNA was extracted from approximately 200 mg of semi-purified product using the E.Z.N.A Tool DNA Kit (D4015–02; Omega Bio-Tek Inc., Norcross, GA, USA) following the manufacturer’s instructions. DNA samples were stored in 200 μl of the kit's Solution Buffer at 20°C until use.

The ITS region of the rRNA was amplified for identification and molecular characterization of *E*. *bieneusi*. ITS-positive samples were further amplified based on MS1, MS3, MS4, and MS7 loci, respectively. The primers and annealing temperatures were previously reported [22]. Secondary PCR products were visualized by staining with Golden View following 1% agarose gel electrophoresis.

### Sequencing and phylogenetic analysis

The amplicons of the expected size were sent to Invitrogen (Shanghai, China) for sequencing. To ensure sequence accuracy, a two-directional sequencing method was used. To determine *E*. *bieneusi* genotypes, the sequences obtained in this study were aligned with sequences downloaded from the GenBank database via BLAST analysis (http://blast.ncbi.nlm.nih.gov) and through the use of ClustalX software. Phylogenetic analysis of ITS sequences was performed using Mega software (http://www.megasoftware.net/), and neighbor-joining phylogenetic analysis of the aligned *E*. *bieneusi* sequences was performed to support genotype classifications. A total of 1,000 replicates were used for bootstrap analysis.

### Statistical analysis

Differences in infection rates among different NHP species and among animals in captivity or the wild were assessed using the chi-square test conducted by SPSS version 17.0 software (SPSS Inc., Chicago, IL, USA). A P-value < 0.05 was considered significant.

### Nucleotide sequence GenBank accession numbers

Representative *E*. *bieneusi* sequences of the ITS gene were deposited in the GenBank database under the accession numbers KX905204–KX905206, and KX905208-KX905211. Similarly, the representative nucleotide sequences of MS1, MS3, MS4, and MS7, obtained in this study, were deposited in the GenBank database under accession numbers KX905213–KX905227 (MS1), KX905228, and KX905231-KX905233 (MS3), KX905234, and KX905236 (MS4), and KX905237–KX905240 (MS7).

## Results

### Occurrence of *E*. *bieneusi* in NHPs

Of 369 fecal specimens from NHPs, four species were present, and 46 samples, with an infection rate of 12.5%, were positive for *E*. *bieneusi*. Specifically, 26 of 303 (8.6%) rhesus macaques and 20 of 30 (66.7%) northern white-cheeked gibbons were infected with *E*. *bieneusi*. However, all short-tailed Tibetan macaques (n = 30) and golden snub-nosed monkeys (n = 6) examined were negative for *E*. *bieneusi*. The difference in infection rates among NHP species was significant (*P* < 0.05). The infection rates of captive and wild NHPs were 14.9% and 1.5%, respectively, and these values were significantly different (*P* < 0.05) (Table 1).

### Genotype distribution and phylogenetic analysis

**Fig 1:**
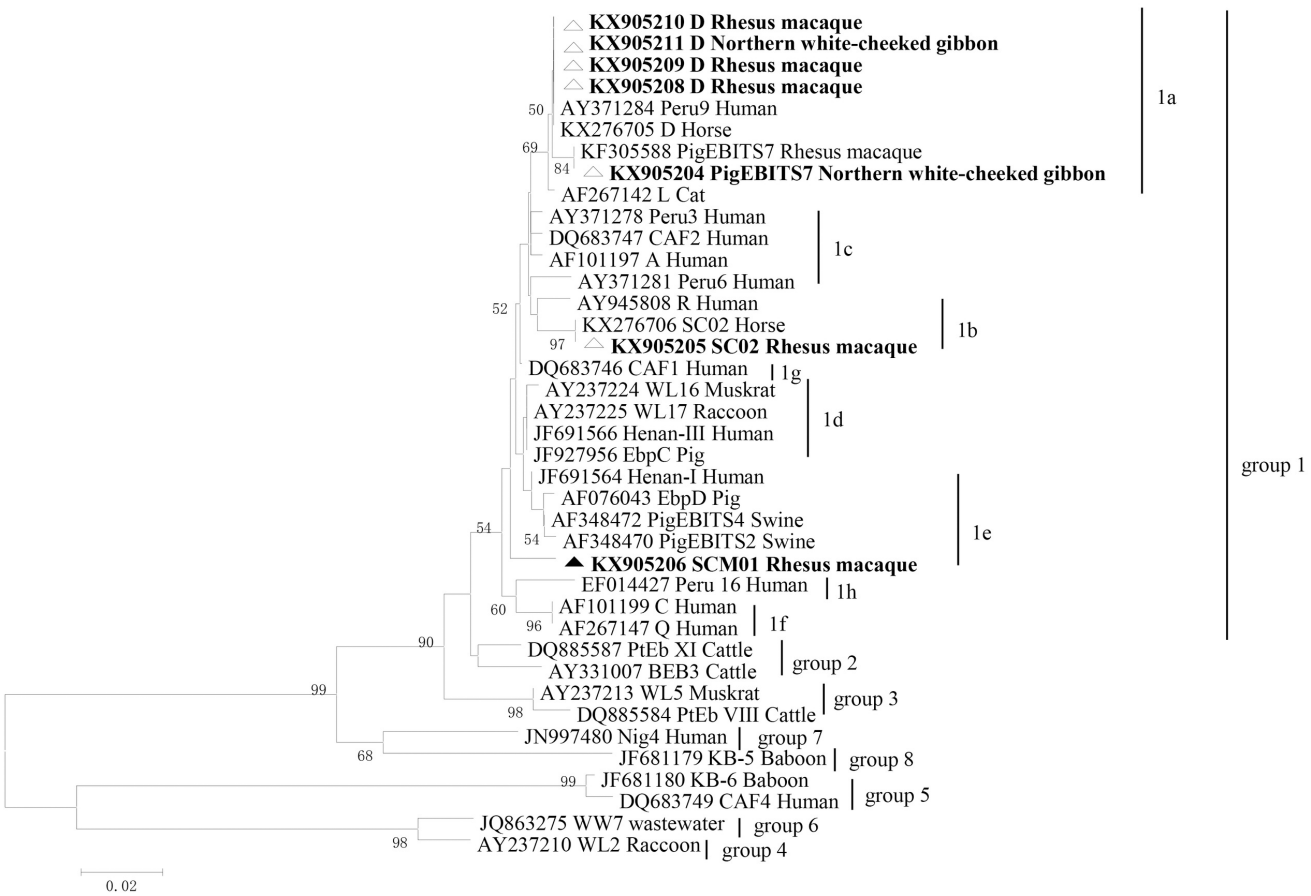
Phylogenetic relationships of ITS nucleotide sequences of the *E*. *bieneusi* genotypes identified in this study and other reported genotypes. The phylogeny was inferred by a neighbor-joining analysis. Bootstrap values were obtained using 1,000 pseudo-replicates and greater than > 50% was shown on nodes. The genotypes in this study are marked by triangles and the novel genotypes are marked by full triangles.

Our study showed the presence of *E*. *bieneusi* in NHPs. Only three known *E*. *bieneusi* genotypes (D, PigEBITS7, and SC02) and a novel genotype named SCM01 were identified. Genotype D was most prevalent (n = 33) and discovered in rhesus macaque and northern white-cheeked gibbon; this was followed by PigEBITS7 (n = 9), which was found in northern white-cheeked gibbon. SC02 and SCM01 both found in rhesus macaque were identified in three samples and one specimen, respectively. With regard to the new genotype SCM01 from rhesus macaques, there were five nucleotide differences compared to AY371278 isolated from patients. Moreover, phylogenetic analysis showed that this isolate formed a new branch (Fig 1).

Phylogenetic analyses based on ITS sequencing showed that all representative isolates detected in our study were distributed into three subgroups belonging to zoonotic group 1 (Fig 1). Genotype D and PigEBITS7 belonged to subgroup 1a, and SC02 clustered into subgroup 1b. SCM01 fell into subgroup 1e.

### MLST genotypes of *E*. *bieneusi*

DNA from 46 ITS-positive *E*. *bieneusi* species isolated from NHPs were further amplified by nested PCR based on three microsatellites (MS1, MS3, and MS7) and one minisatellite (MS4). In total, 36, 37, 30, and 29 specimens were successfully amplified and sequenced at the MS1, MS3, MS4, and MS7 loci, respectively. Moreover, 15, four, three, and five genotypes were formed in MS1, MS3, MS4, and MS7 loci, respectively. Twenty-four specimens were successfully amplified and sequenced at all four loci, which formed 13 multilocus genotypes (MLGs). For ITS genotype D, nine MLGs were characterized. For ITS genotype PigEBITS7, four genotypes were discovered. One MLG was identified for ITS genotype SCM01 (Table 2).

**Table 2:** Multilocus genotypes of *Enterocytozoon bieneusi* from nonhuman primates.

ITS genotype	common name (scientific name)	Multilocus genotypes				
		MS1	MS3	MS4	MS7	MLGs
SCM01	Rhesus macaque (*Macaca mulatta*)	Type2	Type6	Type2	Type2	MLG1
D	Rhesus macaque (*Macaca mulatta*)	Type6	Type1	Type1	Type1	MLG2
D	Rhesus macaque (*Macaca mulatta*)	Type6	Type1	Type4	Type1	MLG3
D	Rhesus macaque (*Macaca mulatta*)	Type5	Type1	Type1	Type1	MLG6
D	Rhesus macaque (*Macaca mulatta*)	Type5	Type1	Type1	Type1	MLG6
D	Rhesus macaque (*Macaca mulatta*)	Type10	Type1	Type1	Type1	MLG4
D	Northern white-cheeked gibbon (*Nomascus leucogenys*)	Type11	Type1	Type1	Type1	MLG11
D	Northern white-cheeked gibbon (*Nomascus leucogenys*)	Type15	Type1	Type1	Type1	MLG8
D	Northern white-cheeked gibbon (*Nomascus leucogenys*)	Type15	Type1	Type1	Type1	MLG8
D	Northern white-cheeked gibbon (*Nomascus leucogenys*)	Type15	Type1	Type1	Type1	MLG8
D	Rhesus macaque (*Macaca mulatta*)	Type10	Type1	Type1	Type1	MLG4
D	Rhesus macaque (*Macaca mulatta*)	Type9	Type4	Type1	Type4	MLG5
D	Northern white-cheeked gibbon (*Nomascus leucogenys*)	Type15	Type1	Type1	Type1	MLG8
D	Northern white-cheeked gibbon (*Nomascus leucogenys*)	Type15	Type1	Type1	Type1	MLG8
D	Northern white-cheeked gibbon (*Nomascus leucogenys*)	Type11	Type1	Type1	Type1	MLG11
D	Northern white-cheeked gibbon (*Nomascus leucogenys*)	Type13	Type1	Type1	Type1	MLG12
D	Northern white-cheeked gibbon (*Nomascus leucogenys*)	Type14	Type1	Type1	Type1	MLG13
PigEBITS7	Northern white-cheeked gibbon (*Nomascus leucogenys*)	Type4	Type1	Type1	Type1	MLG9
PigEBITS7	Northern white-cheeked gibbon (*Nomascus leucogenys*)	Type3	Type1	Type1	Type2	MLG10
PigEBITS7	Northern white-cheeked gibbon (*Nomascus leucogenys*)	Type15	Type1	Type1	Type1	MLG8
PigEBITS7	Northern white-cheeked gibbon (Nomascus leucogenys)	Type9	Type1	Type1	Type1	MLG7
PigEBITS7	Northern white-cheeked gibbon (Nomascus leucogenys)	Type9	Type1	Type1	Type1	MLG7
PigEBITS7	Northern white-cheeked gibbon (Nomascus leucogenys)	Type9	Type1	Type1	Type1	MLG7
PigEBITS7	Northern white-cheeked gibbon (Nomascus leucogenys)	Type9	Type1	Type1	Type1	MLG7

## Discussion

To date, molecular epidemiological studies on *E*. *bieneusi* in NHPs has mainly been conducted in China, owing to the abundance in this country, but studies are still limited. NHPs are reservoirs for *E*. *bieneusi*, with a prevalence of up to 66.7% in cynomolgus monkey [13]. An overall infection rate of 12.5% was observed in our study, which is identical to that in Kenya (12.3%) [18]. In regard to rhesus macaques, 26 of 303 (8.6%) animals were positive, which is lower than the proportions reported in previous studies by Karim *et al*. [1] (31.1% in zoo animals), and Ye *et al*. (28.2% in animals from public parks), but higher than those of Karim *et al*. [13] (8.8% in animals from various habitats). For northern white-cheeked gibbons, the infection rate in Guiyang zoo (66.7%) was higher than that in other zoos with a total infection rate of 35.7% [1]. With regard to short-tailed Tibetan macaques and golden snub-nosed monkeys, we did not find any NHPs that were positive for *E*. *bieneusi*. In accordance with the study of Karim *et al* [1], short-tailed Tibetan macaques were not found to be infected by this organism. There are still no reports of *E*. *bieneusi* infection in Tibetan macaques. However, a 3.5% infection rate was found in golden snub-nosed monkeys in previous study [13]. Our results suggested that captive NHPs were more easily infected with *E*. *bieneusi* than wild NHPs, based on infection rates of 14.9% and 1.5%, respectively. Similarly, the occurrence of *E*. *bieneusi* infection was significantly higher in captive NHPs (13.7%) than in free-range NHPs (5.0%) [13]. The results of these studies might indicate that captive animals are more prone to infection by *E*. *bieneusi* than wild animals. This might be due to the fact that the transmission between NHPs occurs easily for captive animals, as they are housed in relatively narrow spaces and are closer to each other than NHPs in the wild. Furthermore, animal management might play an important role in this transmission of *E*. *bieneusi*.

Currently, fecal samples from more than 20 NHPs such as crab eating macaque, rhesus macaque, golden monkey, olive baboon, blackcapped capuchin, weeper capuchin, white-fronted capuchin, Japanese macaque, and western lowland gorillas have been determined to be positive for *E*. *bieneusi*, and more than 50 genotypes have been described [12, 18, 20, 23]. According to previous studies, D, BEB6, O, EbpC, Type IV, Henan V, Peru8, PigEBITS7, I, CM1, CM4, CM5, CM6, CM7, CM8, Peru11, WL15, LW1d, Macaque1, and Macaque2 have been found in rhesus macaque [1, 13, 23]. However, we only identified D, SC02, and SCM01, with D showing predominance. This result might suggest that D is endemic in the Sichuan province and that *E*. *bieneusi* genotype distribution is relatively narrow compared to that in other areas. Moreover, SC02 and SCM01 were first identified in rhesus macaques. Genotypes Henan-IV, D, and O were described in northern white-cheeked gibbons, whereas our study identified D and PigEBITS7 genotypes [1]. To date, CM4 and D were found in captive golden snub-nosed monkeys, but we failed to identify any golden snub-nosed monkeys infected with *E*. *bieneusi*. This might be due to the fact that the golden snub-nosed monkeys examined in our study were living in the natural environment.

Apart from the new genotype and PigEBITS7, the other genotypes identified in this study have been previously reported in China in various animals, including D in animals of the order Carnivore such as the Siberian tiger, the order Artiodactyla including hippopotamus, the order Primates including orangutans, and the order Psittaciformes including Fischer’s lovebird [24]; SC02 has been identified in Tibetian blue bear, Asiatic black bear, Malayan sun bear, and Northern raccoon [22]; These results together suggest the likely occurrence of cross-species transmission. Furthermore, genotypes D, and PigEBITS7 have been observed in human living in China as well, and genotype D were also found in people from Nigeria, Niger, Vietnam, Cameroon, Gabon, Malawi, Holland, and England [4, 6, 25–30]. NHPs have many biological characteristics similar to humans and are susceptible hosts to some human infectious diseases. Moreover, the *E*. *bieneusi* genotypes identified in our study either have been reported in humans or are considered human-pathogenic genotypes belonging to zoonotic group 1. Thus, zoonotic transmission of *E*. *bieneusi* could happen more easily for these strains. The identification of these genotype further demonstrates that the zoonotic potential and diagnosis of *E*. *bieneusi* using molecular tools has improved our understanding of their transmission to some extent. Whether these *E*. *bieneusi* genotypes are circulating within NHPs population without pathogen introduction from humans or NHPs remains unclear, since NHPs are in close contact with humans. Further studies are needed to fully elucidate the source of NHPs *E*. *bieneusi* infection.

Based on the ITS gene locus, we only identified five genotypes, but multilocus genotyping revealed 13 genotypes, this verifies genetic diversity and further demonstrates that MLST tool has higher resolution for *E*. *bieneusi* than methodology based on one genetic marker. MLG6 and MLG7 were identified in some rhesus macaques and northern white-cheeked gibbons, respectively. MLG8 was found both in rhesus macaques and northern white-cheeked gibbons. These results indicate that interspecies and cross-species transmission probably occurred.

In summary, four genotypes were characterized as having been reported in humans before or were regarded as human-pathogenic genotypes, of which, one novel genotype (SCM01) was identified in a rhesus macaque. Genetic diversity was observed via the MLST tool, wherein 13 MLGs were found in NHPs. This molecular investigation has revealed the common existence of *E*. *bieneusi* in NHPs in southwest China and suggested the potential transmission of *E*. *bieneusi* between humans and NHPs. Thus, deep concerns regarding the importance of NHPs in zoonotic transmission should be raised. Contact between humans and NHPs should be restricted to reduce zoonotic transmission.
